# The Art of Designing DNA Nanostructures with CAD Software

**DOI:** 10.3390/molecules26082287

**Published:** 2021-04-15

**Authors:** Martin Glaser, Sourav Deb, Florian Seier, Amay Agrawal, Tim Liedl, Shawn Douglas, Manish K. Gupta, David M. Smith

**Affiliations:** 1 Peter Debye Institute for Soft Matter Physics, Leipzig University, Linnéstraße 5, 04103 Leipzig, Germany; martin.glaser@uni-leipzig.de; 2 Fraunhofer Institute for Cell Therapy and Immunology, Perlickstraße 1, 04103 Leipzig, Germany; florian.seier@izi.fraunhofer.de (F.S.); amayagrawal22@gmail.com (A.A.); 3 Dhirubhai Ambani Institute of Information and Communication Technology, Gandhinagar 382 007, India; sourav_deb@daiict.ac.in; 4 Faculty of Physics and Center for Nanoscience (CeNS), Ludwig-Maximilians-Universität, Geschwister-Scholl-Platz 1, 80539 München, Germany; tim.liedl@physik.lmu.de; 5 Department of Cellular and Molecular Pharmacology, University of California, San Francisco, CA 94158, USA; shawn.douglas@ucsf.edu; 6 Institute of Clinical Immunology, University of Leipzig Medical Faculty, 04103 Leipzig, Germany

**Keywords:** DNA nanotechnology, CAD software, DNA origami, nanofabrication, DNA bricks, DNA tiles, self-assembly, simulation

## Abstract

Since the arrival of DNA nanotechnology nearly 40 years ago, the field has progressed from its beginnings of envisioning rather simple DNA structures having a branched, multi-strand architecture into creating beautifully complex structures comprising hundreds or even thousands of unique strands, with the possibility to exactly control the positions down to the molecular level. While the earliest construction methodologies, such as simple Holliday junctions or tiles, could reasonably be designed on pen and paper in a short amount of time, the advent of complex techniques, such as DNA origami or DNA bricks, require software to reduce the time required and propensity for human error within the design process. Where available, readily accessible design software catalyzes our ability to bring techniques to researchers in diverse fields and it has helped to speed the penetration of methods, such as DNA origami, into a wide range of applications from biomedicine to photonics. Here, we review the historical and current state of CAD software to enable a variety of methods that are fundamental to using structural DNA technology. Beginning with the first tools for predicting sequence-based secondary structure of nucleotides, we trace the development and significance of different software packages to the current state-of-the-art, with a particular focus on programs that are open source.

## 1. Introduction

DNA plays the central role in the storage and transmission of genetic information in all biological systems. It forms long macromolecules, called single-stranded DNA, which are composed of four different nucleotides (adenine [A], thymine [T], cytosine [C], and guanine [G]), which are connected via a phosphate-deoxyribose-backbone. In 1953, Watson and Crick described the DNA double helix, a secondary structure that formed by two anti-parallel single strands due to base-pairing of the complementary nucleotides A and T or C with G, respectively [1]. Thus, any two complementary DNA segments hybridize into double helices. This assembly depends on factors, such as the salt concentration in the surrounding buffer as well as the temperature, which can be precisely controlled while using a bottom-up approach. Based on this reliable and predictable behavior, in 1982 Nadrian Seeman proposed the idea to use DNA as a versatile material to construct objects on the nanometer scale, pioneering the field of DNA nanotechnology with his groundbreaking work [2].

**Figure 1:**
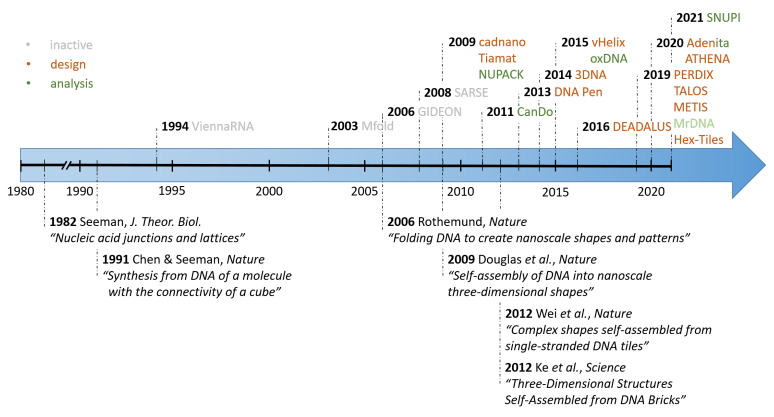
Overview of the rapid development of computer-assisted design (CAD) software (**top row**) alongside with some major experimental novelties (**bottom row**) in the growing field of DNA nanotechnology. The initial release of a few precursor software solutions led to the development of more complex and specialized solutions in the last decade.

One goal of structural DNA nanotechnology is to design artificially programmable DNA nanostructures, generally known as “bottom-up” constructions. Using these methods, complex DNA nanostructures can be made out of double stranded DNA helices [2,3] and employed in various biological applications [4,5,6] as well as for high-capacity data storage [7,8]. structures were initially created using a pen and paper approach, which is time consuming, more error prone, and limits the complexity of the designed structures, since software aided solutions were only made available later on (Figure 1). Nonetheless, during that time complex structures were assembled, for example, Chen et al. reported the first closed polyhedral DNA nano-object, a DNA cube, in 1991, as the first experimental demonstration of Seeman’s vision [9]. Soon after this, in 1995 Erik Winfree showed that the self-assembly of DNA is Turing universal [10], meaning that, in principle, rather than by trial and error, one can systematically design any arbitrary shape or perform any computation using DNA. In 2006, Paul Rothemund, while working in the Winfree lab, reported a remarkable technical advance. He extended the self-assembly of DNA to a more complex dimension by folding the single-stranded genome of an M13 bacteriophage (a virus that infects bacteria) to shapes, such as a smiley face, star, map of north America, and more by using another set of DNA helper strands that work as staples (using the Watson-Crick base pairing) to stably clamp the viral DNA in a specific shape [11,12]. Reminiscent of the Japanese art of folding paper into distinct shapes, Rothemund called his method DNA origami. In order to fold the DNA, he wrote computer scripts to determine which DNA staples would give rise to the specified shape, which was a logical next step due to the large number (approximately 150–200) of involved strands.

While simple DNA-based architectures, such as immobile holiday junctions, wireframe polyhedral, or even larger structures of repeating tiled units, can be designed on-the-fly with pen and paper, this becomes a laborious process when moving up the ladder of complexity to more sophisticated designs consisting of many more motifs and unique sequences. As an example, creating a precise design for one of the most conceptually simple two-dimensional DNA origami structures, the so-called Rothemund Rectangle, involves the linking of 171 four-way Holliday junctions, and the assignment of 6912 bases according to the sequence of the underlying scaffold and its topological path through the overall structure. More intricate designs that are based on this or other methods drastically complicate the process, particularly since this requires the daunting task of visually mapping the three-dimensional network of interconnected junctions onto a two-dimensional schematic.

Therefore, computer-based solutions are needed to construct increasingly complex structures. Initial tools for analysis have been around since the early ‘90s, such as ViennaRNA [13]. However, this solution was solely limited to computing secondary structures of RNA by the minimization of the free energy. The first available computer-assisted design (CAD) software package to actually design a DNA nanostructure de novo was GIDEON, which was developed by the group of Ned Seeman in 2006 [14]. Around this time, several other initial software solutions were also developed, like SARSE and UNIQUIMER 3D [15,16]. However earlier CAD software often required a certain level of computational skills and a priori knowledge of DNA-based design, thus limiting the accessibility to the entire DNA nanotechnology community. Nevertheless, a steady stream of CAD software packages for designing different types of simple and complex DNA nanostructures has emerged with increasingly user-friendly graphical user interfaces and abilities for creating complex designs from scratch. Whether by coincidence or otherwise, the same timeframe has also corresponded to a rapid expansion of the DNA nanotechnology from being an interesting niche oddity, to making inroads in fields from nano-photonics to medicine and healthcare. For these applications, the DNA origamis are of particular interest, since they allow for the precise positioning of molecules and structures for specific applications [17,18].

Therefore, this review covers some of the most important milestones in the rapid development of semi- or fully automated design software for both: either scaffold-based DNA or tile-based DNA structures, pointing out their applicability for an anticipated application. We mostly focus on programs that enable the user to initially envision designs based on abstract geometrical forms, and generate the sequences of oligonucleotides necessary for assembly, rather than from the starting point of existing DNA motifs. Nevertheless, we do briefly describe some of the currently available analytical tools for DNA nanostructures, and briefly conclude with some recently developed tools that point towards the future generation of all-in-one software suites.

## 2. Scaffolded DNA Origami

**Figure 2:**
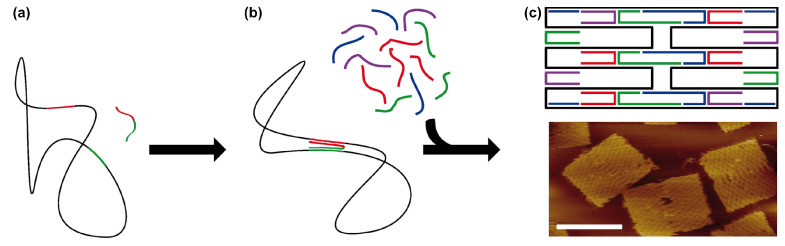
Principal formation of a DNA origami. (**a**) The scaffold strand functions as a backbone and is clamped together by a set of staple strands. (**b**) Representation of a small rectangle. (**c**) Atomic Force Microscope (AFM) image of a typical structure of dimensions 100 × 70 nm (scale bar: 50 nm). Picture adapted from Smith et al. [4].

The DNA origami technique was first introduced by Paul Rothemund in 2006 [12], and it is largely responsible for the rapid expansion of the DNA nanotechnology field from a mostly niche area of research to its current status as an integral tool for a broad number of areas. In the 15 years since its first introduction, many different design strategies for creating DNA origami structures have been introduced, including densely packed two- and three-dimensional structures based on parallel helices [12,19,20], structures containing precise twists and curves [21], thin-edged wireframes [22,23,24,25], geometrically-inspired polyhedra [26,27,28], enclosed boxes [29], structures that are folded and cut into topological architectures [30], pre-stressed tensegrity structures [31], and countless others. At their root, all of these specific design implementations share a common underlying strategy: a long, single-stranded, so-called “scaffold strand”, which typically consists of several thousand bases of a known sequence, is folded and stably clamped into a specific shape by a collection of several hundred shorter “staple” oligonucleotides (Figure 2). Convenience has typically limited the maximum size of DNA origami structures, since the most commonly-used scaffold strand is derived from the genome of the M13 bacteriophage, which is around 7000 bases in length. These discrete size limits that are imposed by this choice of scaffold (typically several hundred nanometers for a thin rod or approximately one hundred nanometers for a more rigid block) have been increasingly circumvented by using, or genetically modifying, longer scaffold strands [32,33], or alternatively binding together multiple structures into a large aggregate structures [34,35,36,37,38].

When considering the numerous and diverse strategies for architecturally designing a DNA origami structure, there is no one-size-fits-all software package that is suitable for every possibility. Rather, a collection of CAD programs, each of which fits a specific niche in design methodology, have gradually filled the space over the last decade. The earliest and still most commonly used DNA origami structures consist of interconnected, parallel DNA double-helices, which are arranged in either a honeycomb or square lattice, when viewed along the helical axis. Already in 2009, a pair of CAD software packages—Cadnano and Tiamat—were released, which eliminated the need for either manual design or self-written scripts. A comparison of the software packages covered within the section are summarized in Table 1 at the end of this section.

### 2.1. Cadnano

**Figure 3:**
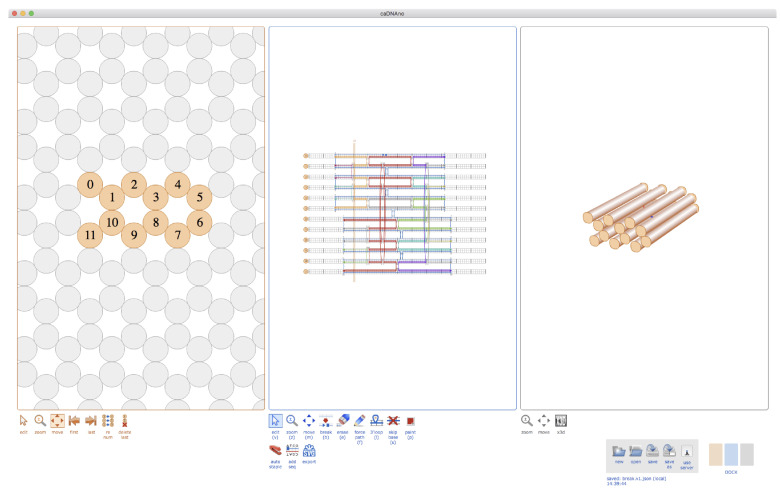
The original Cadnano interface. On the left, the “Slice” view shows abstracted orthographic view of the helix lattice. Empty gray circles represent positions within the lattice where helices can be added to the design. Using keyboard and mouse controls, the user can add DNA helices to the data structure. Active helices are colored orange and assigned a helix number. In the middle view, the “Path” panel provides a two-dimensional schematic blueprint of the DNA origami scaffold and staple paths, which are overlaid on grid squares that represent single nucleotides. On the right, the three-dimensional view displays a crude three-dimensional rendering of the shape in which helices are represented as cylinders that are capped with circular endpoints.

Cadnano [39] was developed as an open-source CAD tool to enable the rapid prototyping of two- and three-dimensional DNA origami nanostructures via a user-friendly graphical-user interface (GUI). Figure 3 provides an overview, also showing the basic design steps. Cadnano supports lattice-based architectures in which the helices are arranged in a honeycomb or square pattern when viewed as a two-dimensional cross-section whose plane is normal to axes of the helices. The initial release was developed by Shawn Douglas in William Shih’s group at Harvard. Subsequent releases (versions 2 and 2.5) were co-developed with Nick Conway at the Wyss Institute, and the software is now maintained by the Douglas Lab at UCSF. DNA origami shapes can be created in a user-friendly process by just using a mouse and keyboard to interact with the GUI. First, the cross-section of the target desired shape is created in the lattice view. Next, one or more single stranded DNA scaffold routes are “drawn” in the path view (Figure 3) and edited with single-base resolution to match the length of a corresponding scaffold stock in the lab. Precursor staple strands are typically added in one step with default anti-parallel crossovers using the “auto-staple” button. Subsequently, staple precursors are edited in order to create desired 5’ and 3’ endpoints using the “break” tool. Once a design is finalized, the staple sequences can be exported to a text file or spreadsheet for ordering. Cadnano relies on open-standard file formats; origami designs are saved as JavaScript Object Notation (JSON) files, and two-dimensional schematics can be exported in Scalable Vector Graphics (SVG) format. Cadnano 2.5 features a Python API which allows for scriptable import and modification of designs. Recently a scriptable, browser-based CAD tool was introduced, referred to as ‘scadnano’, which mimics many of the features of Cadnano, however with added compatibility for Python-based automated scripts, and the ability to more easily design a broader range of DNA structures beyond origami [40].

### 2.2. Tiamat

Tiamat [41] is a GUI platform for the molecular modelling of lattice and scaffold free DNA nanostructures. The tool was developed in 2009 by the research unit of Hao Yan from Arizona State University. Tiamat can be used to visualize and edit DNA nanostructures, and it has an added feature of a DNA sequence generator in its workings module. Unlike a previous DNA sequence generator, SEQUIN [42], Tiamat takes the randomness factor into account. The sequence generator also adds reliability to the tool, as it avoids secondary structures. This software also recognizes different factors, such as uniqueness on subsequence existence, restriction on homopolymer runs (symbol repetitions), and GC-content constraint.

The GUI of Tiamat is structured into three main elements. On the top is the toolbar; the middle part is split into four panels, each representing a different perspective of the three-dimensional space, used as working areas; on the bottom, the current three-dimensional position of the cursor and the size of the currently selected DNA helix are displayed. By activating the tool to create DNA strands, helices can be added in each of the four panels of the middle part. In the following pop-up window, further specifications of the entered DNA strand can be set, such as the precise length, its 3’ and 5’ orientation, whether the DNA is single or double stranded, or whether a random sequence should be assigned. Through various other tools, each nucleotide can be selected and modified, e.g., crosslinks between two helices can be manually added. Tiamat also has tools to enable adding specific sequences, selecting individual parts, creating free DNA loops, and so on.

### 2.3. vHelix

**Figure 4:**
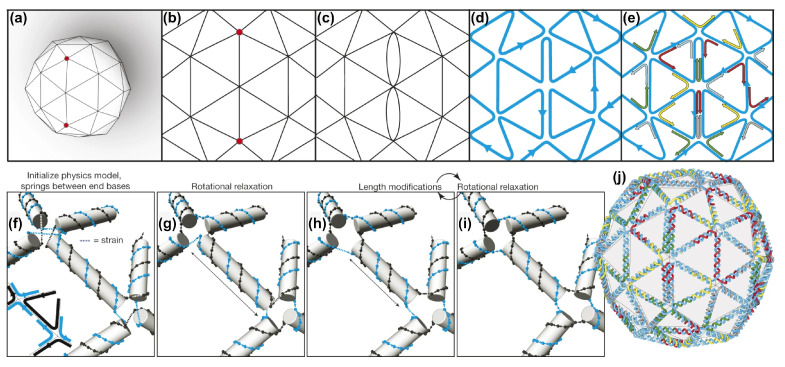
Example of scaffold routing and strain relaxation in vHelix. (**a**) Using virtually any shape as input, a three-dimensional mesh can be generated based on a scaffold routing through individual vertices. (**b**,**c**) To eliminate odd vertex numbers double edges are introduced. (**d**,**e**) The A-trails algorithm routes the scaffold (blue) through the structure and staple-strand (multi-coloured) are included. (**f–i**) Before the final output (**j**) a routine is applied to minimize the remaining internal stress. Reproduced with permission from [24]. Copyright Springer Nature Ltd., 2015.

vHelix is a plug-in for the commercially available three-dimensional modelling software Autodesk Maya, that can be used for the design of wireframe DNA nanostructures [24]. It was developed by the Högberg Research Group at Karolinska Institutet in Stockholm. The software uses a specialized approach focusing only on polyhedral meshes, which is a mesh enclosing a volume with an arbitrary number of vertices, edges, and faces (Figure 4). These structures can also be designed with previously existing software, but they would need manual adjustments that arise from geometrical constrains of the DNA and sense/antisense pairing, which are automated by this approach. Furthermore, previous approaches usually folded the circular single-stranded scaffold DNA into a tree-like shape, and connected segments via so-called “helper joins”, which are short single DNA strands forming links between two disconnected “blunt end” scaffold loops. However, this design method for wireframe meshes would require hundreds of these helper join strands per structure, which lead to aggregation problems [24]. The main advantage of this method is that the algorithm optimizes the path of the scaffold in a way that it transverses every edge once. Mathematically, this is closely linked to the ‘route Inspection problem’ in graph theory, which goes back to an old question that is known as ‘the Seven bridges of Königsberg’ [43]. The problem revers to the question: if seven bridges connect the central part of the city with the three surrounding parts—is it possible to visit all four parts of the city and cross each bridge only once? It was shown by Euler that no such route exists [44] and that in a more general sense such loop walks require an even degree at each vortex, edges in the case of wireframes. In vHelix, after drawing the desired mesh with Maya, the algorithm pairs odd-degree vertices (in a certain proximity) in order to eliminate them by introducing double edges (Figure 4b,c). This allows for the scaffold to travel through any desired wireframe structure, which is then brought into the final shape by staple strands. The algorithm finds this routing with a very short computing time. Before the final output, torsional strain is also equally distributed in the structure by the software. The main advantage of this construction technique is that most three-dimensional shapes can be broken down into polygon meshes, particularly made up of triangular tiling, which have a high rigidity, and these can now be implemented despite having an odd vertex degree. Furthermore, the final outcome only consists of DNA double helices, which circumvents the problem of unphysiologically high salt concentrations that are necessary for closed-packed bundles of helices that are found in most previous DNA origami designs.

### 2.4. DAEDALUS, PERDIX, TALOS, METIS

**Figure 5:**
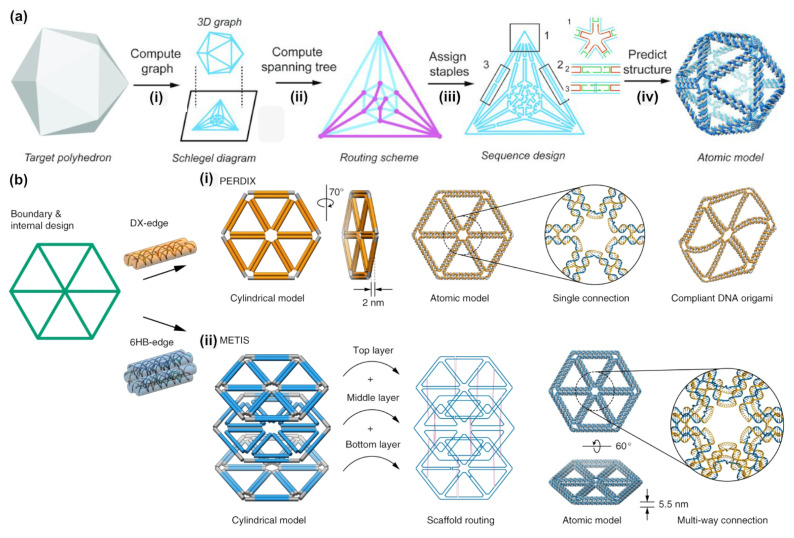
Automated routing and sequence generation for a user defined mesh according to DAEDALUS. (**a**) (i) Starting from the input a three-dimensional graph and its Schlegel diagram is calculated. (ii) Computation of the spanning tree, followed by the scaffold routing. (iii) Assigning the needed staple sequences. (iv) Output of the atomic model. (**b**) Difference in the design of a two-dimensional wireframe scaffolded DNA origami objects with DX or 6HB edges. (i) Each wireframe edge is connected to its neighbor via one scaffold and one staple crossing. (ii) To form more stable structures the algorithm uses three layers connected with scaffold double-crossovers. Each wireframe edge is connected to its neighbor via three scaffold and one staple crossings. (**a**) Reproduced with permission from [28]. Copyright The American Association for the Advancement of Science, 2016. (**b**) Reproduced with permission from [47]. Copyright Springer Nature Ltd., 2019.

These of four software programs are closely related to each other and they focus on the design of scaffold-based DNA wireframe structures. They have been developed and are maintained by the Laboratory for Computational Biology & Biophysics at MIT directed by Prof. Mark Bathe. The connection is also indicated by their according acronyms, each of which refers, in a more or less sophisticated manner, to a figure from Greek mythology. The first program of this group, DEADALUS, was released in 2016 [28], and it is freely available as a MATLAB or Python source code. DAEDALUS is specialized for creating DNA origami renderings of polyhedral networks. The desired shape can either be directly entered into the software by assigning the vertices, connecting edges and corresponding faces, or input through a number of CAD file formats that specify polygonal geometry (e.g., PLY, STL, or WRL format). This software then calculates a two-dimensional representation of the three-dimensional object with the scaffold DNA running through this entire tree (Figure 5a). The staple strands are chosen in a manner that interconnected edges consist of two duplexes that are joined by antiparallel double crossovers. One of the main advances of this approach is that the desired shape does not need to be topologically equivalent to a sphere, which broadens the possibility for more arbitrary architectures (e.g., a torus, whereas vHelix assembled a nicked torus) [28]. The software provides the full set of staple strands for either a given scaffold strand by the user or it generates a default scaffold strand. Additionally, it returns a PDB (“Protein Data Bank”) file, which contains a complete three-dimensional structural model down to the atomic scale.

Following this software, several more specific programs that were suited to specific design strategies were also made available. PERDIX overcomes the limitation of DEADALUS in rendering two-dimensional objects. It uses a similar approach, but it can now account for planar geometries and arbitrary network edge lengths and vertex angles [45]. It also requires a CAD input file and returns a list of the staple strand fitting to the scaffold strand. The source code is freely available in Fortran. Later, TALOS was developed, which uses this approach to construct three-dimensional objects with edges that are based on “6-helix bundle” (6HB) designs [46]. As a further addition, METIS was established to specifically generate two-dimensional wireframe origami by stacking three layers on top of each other (Figure 5b) [47]. Choosing 6 HB edges over double crossover edges significantly increases the mechanical stability due to the higher number of involved helices at each edge, thus giving it a broader applicability.

All of the described solutions have recently been incorporated into one platform, called ATHENA [48]. It provides the user with an intuitive GUI, which drastically increases the easy usability of each of the previous algorithms, so that any wireframe DNA origami in two- or three dimensions using either 2 HB or 6 HB edges can be created.

## 3. DNA Tiles and DNA Bricks

**Figure 6:**
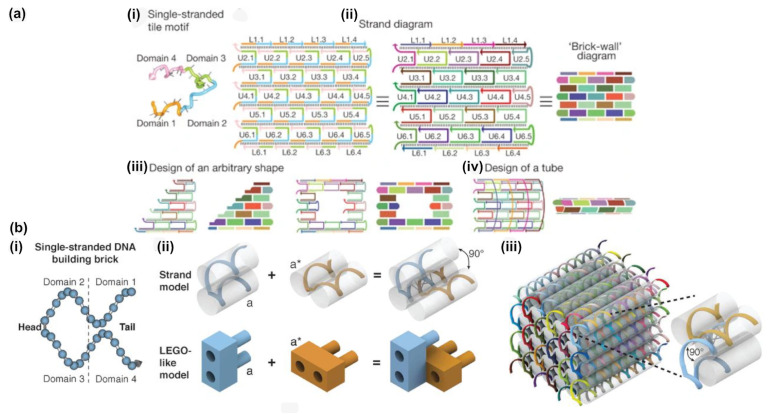
The assembly of DNA tiles and DNA bricks. (**a**) (i) The original single stranded (full) DNA tile is 42 nt long. Conceptually it is divided into four domains, which are partially combined to form a given structure. (ii) Design of a simple rectangle. (iii) By a clever choice of the single strands more complex shapes like a triangle (left) or a rectangular ring (right) can be achieved. (iv) Choosing self-overlapping sheets allows for the creation of hollow tubes. (**b**) Design of DNA brick structures similar to LEGO® bricks. (i) The basic building block is a 32 nt long single DNA strand with four domains, each is 8 nt in length. Domains 2 and 3 are called “head” domains, domains 1 and 4 are called “tail” domains. (ii) Two-bricks assemble via two complementary 8-nt domains with a 90° angle. (iii) A molecular model of a simple cuboid DNA structure. (**a**) Reproduced with permission from [49]. Copyright Springer Nature Ltd., 2012. (**b**) Reproduced with permission from [50]. Copyright The American Association for the Advancement of Science, 2012.

In 2012, two so-called scaffold-free methods for the DNA-based nanofabrication of discrete structures were introduced in rapid succession: DNA tiles and DNA bricks [49,50]. In contrast to DNA origami design methods, these strategies rely on the Lego-like assembly of hundreds or even thousands of shorter synthetic oligonucleotides into objects of pre-defined shape and size (Figure 6). The two-dimensional tile method uses a similar topological scheme to earlier single-stranded tile-based assembly [51] to create single-layer, sheet-like structures, with nearly any shape available within a molecular canvas. DNA Bricks go one step further, exploiting the helical nature of double stranded DNA to create three-dimensional structures on a square or honeycomb lattice, identical to those that were used for DNA origami.

When compared to the DNA origami technique, the DNA brick strategy in particular has the advantage of not being constrained by the number of base pairs in the underlying scaffold strand, or its typically circular topology. It is also straightforward to create arbitrarily large structures either by simply using an expanded set of component oligonucleotides bricks [52], or by using a repetitive design that assembles into mesoscopic crystalline surfaces [53]. Finally, since all individual components are short, synthetically produced oligonucleotides, it is possible, by direct chemical synthesis, to create a denser matrix of functional elements than origami can create, where 50% of the structure is comprised of the more difficult to modify central scaffold strand.

These features make this method an attractive strategy for industrial nanofabrication, where nanoscale precision over distances approaching wafer sizes are invaluable. Indeed, one of the most promising studies based upon this technique demonstrates unprecedented long-range spatial control over the placement of carbon nanotubes, pointing towards a potential real-world future in nano-circuitry [54].

Nevertheless, the application of DNA bricks in research or practical applications has hardly moved the needle in comparison to DNA origami, and it largely remains a niche technique most frequently used to study addressable self-assembly [55,56,57,58,59,60,61,62,63,64,65]. The hurdles to its wider dissemination are two-fold:

1. While offering the aforementioned design advantages, the lack of a central scaffold strand to template the assembly means that the self-assembly process is dependent upon a nucleation-and-growth mechanism [65]. Here, the local structure, topological connectivity, kinetic traps, and even stoichiometry between the hundreds or thousands of components are critical parameters and each can impact yields.
2. Particularly for three-dimensional bricks, the process of translating an arbitrary design into a collection of hundreds or thousands of unique DNA oligonucleotide sequences is extraordinarily complex. The target structure is first rendered as a collection of voxels, each corresponding to an eight base pair segment of double stranded DNA, then connections between the voxels under the constraints of DNA geometry are applied, before each strand is populated with appropriate sequences, according to the original report from Ke et al. [50].

A combination of commercial CAD software, such as Maya or Strata and custom scripts, are typically used, thus imposing a significant barrier to the majority of users, in order to execute the complex design process to create de novo architectures. The aforementioned Tiamat program [41] can, with great effort, be repurposed as a rudimentary program for designing simple tile-based constructs [66]. However, notably, no all-in-one design suite equivalent to Cadnano in terms of built-in features and corresponding experimental validation exists to date. Nevertheless, we will briefly discuss early-stage design packages for this type of design strategy that are available to the public and will be summarized in Table 2 at the end of this section.

### 3.1. Two-Dimensional Tiles with DNA Pen

The software tool DNA Pen [67] is a GUI that can create two-dimensional objects using the modular canvas-based design variant of DNA tiles first introduced by Wei et al. in 2012 [49]. In this specific tile implementation, a typical ‘full’ tile is a DNA oligonucleotide that consists of 42 bases, subdivided into four domains. Each of these subdomains is hybridized with a neighboring tile with 10 or 11 base pairs, which, on average, accounts for a full helical turn. The average of 10.5 bases per subdomain minimizes the internal torque of the assembled constructs. The targeted structure is formed by treating each 42-base brick as a pixel on a two-dimensional canvas, with connectivity between the individual subdomains following the staggered placement of the 42-base tiles. Edges of the structure are typically compensated by “half-tile” sequences or “impervious full-tile sequences”, which partially consist of a poly-T chain to prevent base-stacking where no specific binding is required.

**Figure 7:**
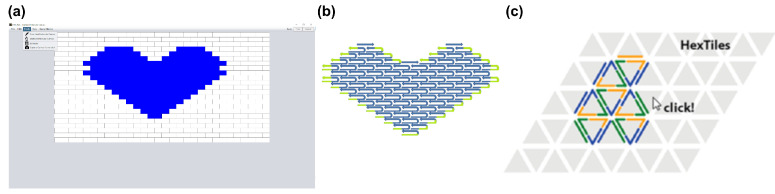
Interface of DNA Pen and Hex Tiles. (**a**) Digitalized molecular canvas representing a heart in DNA Pen. (**b**) Molecular representation of the involved DNA sequences given by the program. (**c**) The user is presented with a virtual canvas in Hex tiles. (**c**) Reproduced with permission from [69]. Copyright American Chemical Society, 2019.

The fundamental new idea behind the DNA pen is to allow the user to freely draw a molecular canvas by using the cursor as a paint brush. The tool will then assign a fit of pixels to the drawing, and the corresponding pattern of tile oligonucleotides, after which the according sequences can be calculated. Alternatively, the user can also choose a digitalized molecular canvas, whereupon they choose pixels (each of which directly corresponds to a 42-base tile oligonucleitide) along the horizontal and vertical axes to construct digital nano objects of the desired shape (Figure 7a). After completing the basic design, the user can assign and output sequences to the tiles in a separate .csv file, either as a list of oligonucleotides or with the coordinates for each of the four tile subdomains within the structure given. The structure is also represented in a molecular representation, showing the DNA sequences (Figure 7b). Furthermore, edges that are comprised of “half-tiles” of only 21 bases are appended with poly-T segments in order to prevent base-stacking of blunt ends, which is known to (and sometimes utilized for) multimerize multiple DNA structures [68].

We would like to point out that the term “DNA tiles” is a rather general term that is commonly used to describe two-dimensional, scaffold-free structures that are comprised of interwoven oligonucleotides. The canvas-like variant that is described above is based on the simplest implementation of using topologically repeating single-strands in a U-shaped motif, albeit with unique sequences and addressability, as the basic tile. This was originally developed within the context of polymer-like DNA nanotubes [51,70,71], which have been used for generating macromolecular structures, such as viscoelastic hydrogels [72] and patterned condensates [73]. The earliest envisioning of tiles created from synthetic DNA oligonucleotides were consisting of a “double-crossover” scheme [74], and they were often used for creating large arrays [75,76,77] or nanotubes [78]. While these repeating motifs were simple enough to be designed by hand, some of the CAD programs, such as Tiamat or even Cadnano, could be used in a way to assist in their design. A recent tile-based construction method for creating triangulated wireframe structures from single-stranded tiles also utilized the modular, molecular canvas approach in coordination with a specialized CAD program, called Hex-Tiles (Figure 7c) [69]. Within the program, the user can select the desired pattern on a virtual canvas, which is already tiled to fit the triangulated motif. Oligonucleotides for the core and edges of the structure are generated to fit the pattern, and according sequences are assigned.

### 3.2. Three-Dimensional Bricks with 3DNA

The software platform 3DNA [79] was developed to model, edit, and visualize complex three-dimensional brick-based structures, using the strategy that was introduced by Ke et al. in 2012 [50], and later expanded to larger structures by Ong et al. in 2017 [52]. The conceptual approach to this design strategy is that every voxel in the abstract structural representation corresponds to a double-stranded, eight base pair domain interaction (between different 32 bases long DNA bricks), which defines a voxel size to dimensions of approximately 2.5 × 2.5 × 2.7 nanometers. Each oligonucleotide brick, typically 32 bases in length, except for on the structure boundaries, spans four voxels, and interacts with four other unique bricks. When compared to the aforementioned workflows involving commercial CAD software and self-programmed scripts, 3DNA can increase the ease and accuracy of designing DNA sequences for model-specific formations, as well decrease the time that is needed for these processes.

**Figure 8:**
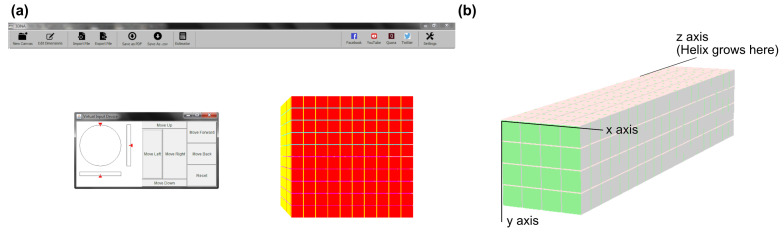
The interface of 3DNA. (**a**). The program lets the user freely rotate a default cuboid. (**b**) By adding or removing single voxel with your cursor any desired shape can be created.

The GUI of 3DNA provides an intuitive environment, even for new users, since voxels, which are represented as cubes in the three-dimensional in-program model, can be easily added and removed from the desired shape (Figure 8a). DNA sequences can be fetched either by considering predefined set of sequences or by directly generating them from the random sequence generator. In particular, the default randomness in unique DNA sequences includes a GC content of 40–60% and a Hamming distance (the number of places where two sequences differ) that is less than or equal to six per domain. In addition, the three-dimensional canvas can be expanded to any preferred dimension in x, y, and z, in order to enable arbitrarily large structures (Figure 8b). Once a desired shape has been modeled within the GUI, the visualization module helps in the further analysis of the structure by providing three different options, i.e., elementary, planar, or full-canvas visualizations. Again, the software provides the user with a full list of all required DNA oligoucleotides, even a cost estimator that is based on approximate per-based nucleotide prices is included. Currently, 3DNA only accounts for the eight base pair voxel structure that was originally introduced by Ke et al. for a square lattice [50], and it does not yet have features for a hexagonal brick structure [80,81,82] or the expanded 13-base-pair voxel introduced later by Ong et al. [52]. The authors of this study created a browser based software to also create and manipulate three dimensional cuboids, a page that is still under ongoing development [83].

## 4. Analysis

Finally, we give brief attention to a variety of publicly available analytical tools that are used for making predictions about the final structure, interactions, or other features of pre-defined, nucleotide-based constructions. A summary of the covered software tools can be found in Table 3 at the end of this section. While this deviates slightly from the main topic of this review, these tools have nevertheless emerged as useful companions in the design process for making approximate *in silico* studies of behaviors under specific conditions.

**Figure 9:**
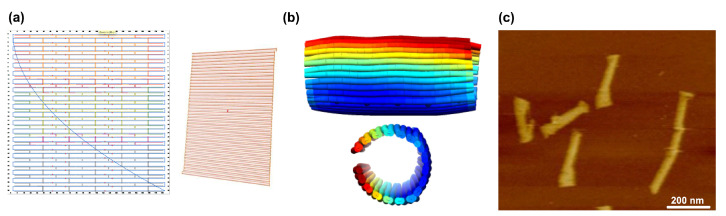
Structural faults in a topologically robust DNA origami design. (**a**) Original design of a single-layer DNA origami sheet in Cadnano, consisting of 39 parallel helices of approximately 180 base pairs in length. (**b**) Physical modelling with analysis tool CanDo indicates a cigar-like roll up of the design, due to accumulated torque between double-helix rows. (**c**) Verification of the undesired global “rolling” deformation with AFM.

Even though they often require too much computation power and/or time to be feasible for direct integration into standalone CAD software, like those described above, these tools are valuable to researchers for the preliminary validation of a particular design, made before incurring the cost of purchasing several hundred or more oligonucleotides. Indeed, even the best *in silico* simulations of a complex DNA origami or brick structure are not equivalent to rigorous experimental confirmation by agarose gel electrophoresis, electron microscopy, or atomic force microscopy. Nevertheless, they can often tip the designer to some subtle problems in the design, which can ultimately mean doom for the self-assembly of the structure. DNA-based CAD software typically accounts for generating suitable topology, connectivity and sequences of the constituent DNA strands for the chosen structure. This ignores the finer thermodynamic or mechanical impacts of certain design motifs, which can additively lead to global faults when summed over the entire structure. These faults can arise from a number of easily overlooked factors, such as, for example, a high local density of short hybridized segments between crossovers [84], or small amounts of torque built up over broad parallel arrays of double-helices. These can lead to structural instability of the structure stemming from local dominance of the self-repulsion between neighboring double-helices, or unwanted global deformations, as shown, for example, in the sheet-like structure shown in Figure 9.

In some cases, multiple, sequence-dependent isoforms can also arise from a single topological design [85]. Therefore, we briefly highlight several freely available simulation and modeling tools for DNA nanostructures, which can potentially aid in the design process.

**Figure 10:**
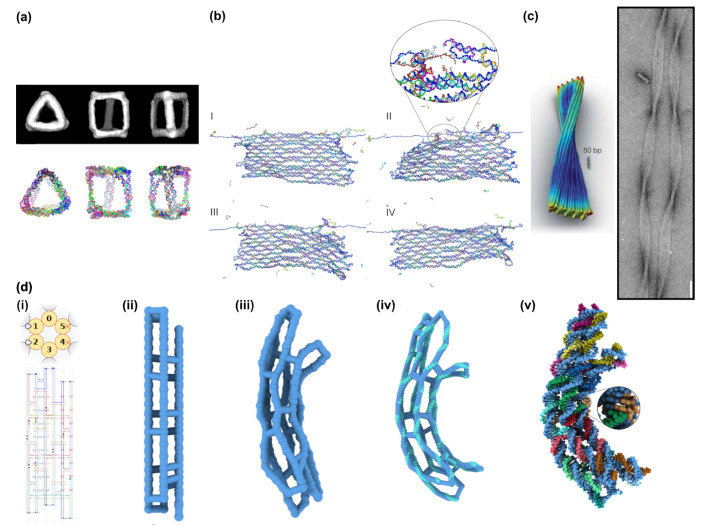
Overview of analytical tools. (**a**) Reconstructed three-dimensional maps of prisms via cryoTEM imaging. Configurations predicted by the oxDNA algorithm, showing typical arrangements of DNA prisms. (**b**) oxDNA simulation of the force-induced unzipping of a DNA origami sheet if the scaffold is pulled from both sides. (**c**) Cando simulations predicts the handedness of a 60 HB bundle object in honeycomb lattice packing where insertions are added to create an effective underwinding to 11 bp per turn correctly, as confirmed by direct TEM imaging (scale bar is 20 nm). (**d**) Structural relaxation of a curved DNA origami object. (i) Cadnano design of a curved six-helix bundle. Insertions (blue loops) and deletions (red crosses) induce the curvature. (ii,iii) Low-resolution coarse-grained simulation of the bundle deformation within 40 ns. (vi) High-resolution coarse-grained simulations of the curved bundle including a local representation of each base pair’s orientation. (v) Resulting atomic model of the curved six-helix bundle. (**a**) Reproduced with permission from [96]. Copyright American Chemical Society, 2016. (**b**) Reproduced with permission from [98]. Copyright American Chemical Society, 2018. (**c**) Reproduced with permission from [90]. Copyright Springer Nature Ltd., 2011. Reproduced with permission from [21]. Copyright The American Association for the Advancement of Science, 2009. (**d**) Reproduced with permission from [101]. Copyright Oxford University Press, 2020.

1. Mfold was released in 2003 on its own web-server-based application and it is one of the oldest for computational molecular biology [86]. On this web server, several analysis tools for sequence analysis and the prediction of RNA and DNA folding can be found, including the Mfold software. In 2008, it was renamed to UNAfold. Mfold and UNAfold anticipate the folding of DNA and RNA strands through the prediction of the structure’s minimum free energy Δ G [87].
2. Nucleic Acid Package (NUPACK) is a design and analysis tool for the base pairing of one or multiple DNA sequences, released in 2010 [88]. The program is suitable for the design of nucleic acid sequences and their thermodynamic analysis. Therefore, it can be used for the evaluation of simple nucleic acid systems.
3. Vienna RNA originally was released in 1994, providing a tool for the prediction of RNA secondary structures [13]. In 2011, the ViennaRNA software package was launched, including technical updates to the GUI and the underlying RNAlib. New tools for the assessment of RNA–RNA interactions as well as additional output information were added [89].
4. CanDo (Computer-aided engineering for DNA origami) is a finite element modeling framework developed at MIT [90]. Originally, it was limited to model honeycomb and square lattice DNA assemblies that were designed using Cadnano, already proving its predictive power (Figure 10c). Later, it got reworked to model wireframe structures allowing for highly complex three-dimensional geometries and their flexibility that would be infeasible analytically [91]. It was later extended to enable lattice-free modelling [92] as well as long time-scale dynamics of DNA assemblies using Brownian Dynamics [93]. Later, CanDo launched its own online server, which even makes modelling to the atomic scale possible [94,95].
5. OxDNA is a simulation code from the University of Oxford that implements a coarse-grained DNA model [96]. The code uses Monte Carlo and Molecular Dynamics simulations for determining the mechanical and thermodynamic properties of single- and double-stranded DNA and RNA (Figure 10a). Taking major and minor grooves into account and by adjusting the coaxial stacking and backbone-backbone interactions, it allows for more precise prediction of especially larger (kilobase-pair) structures. It has been reworked to OxDNA2, which allows for the adjustment of salt concentrations, and treats the interaction of consecutive adenine bases to consecutive thymine bases differently, a feature especially important in systems with flexible single-stranded regions [97]. This model can also be used to predict the involved forces when unraveling a DNA origami by force-induced melting, and it has been experimentally verified via Atomic Force Microscope (AFM), making it an interesting example for the flexibility of this software [98]. A recently-developed web browser-based visualization tool, oxView, provides a fast and user-friendly interface to the underlying code, and it includes additional modules for characterizing aspects, such as structural [99], and a tool, TacoxDNA, is also available for converting common CAD output files (e.g., from Cadnano or Tiamat) into representations that are suitable for simulation via OxDNA [100].
6. Being released in 2019, MrDNA is a tool for the prediction of the structure and dynamics of DNA based systems [101]. The software features a fast multi-resolution model for the prediction of self-assembled DNA origami on an atomistic level in 30 min. or less. This allows for fast in situ simulations and saves a lot of time in a de novo design of complex DNA nanostructures.
7. Finally, the newest addition to the pantheon of analysis tools, SNUPI (Structured NUcleic acids Programming Interface) renders near-atomically-precise analysis of DNA origami properties, such as shape, dynamic properties, and mechanical properties rigidity in a relatively quick processing time [102]. SNUPI functions as a standalone, downloadable program, and acts as a convenient companion tool for visualizing and analyzing structures that are designed within Cadnano. As input, standard JSON files along with an optional sequence file (in .csv format) for the scaffold are used. The analysis engine combines known, intrinsic properties of DNA molecules with sequence-specific geometric and mechanical properties that are determined by molecule dynamics simulations. Subsequently, this information is fed into a structural model to efficiently generate atomic-level information in a matter of minutes.

As is the case with many of the aforementioned CAD tools, the technical development for analysis tools is, of course, ongoing and it aims for increased user-friendly handling and reduced computational time. While, for example, OxDNA needs about two days of running time to simulate a DNA origami structure of several thousand bases, newer software releases, such as SNUPI, provide near to atomic resolution of DNA structures in as little as 15 min.

## 5. Discussion

During the last four decades, the concept of building nanometer-precise materials from DNA strands has advanced from being a crystallographer’s theoretical idea [2] to forming an integral tool for a wide number of fields in basic and increasingly applied research. In the early years, it was a major accomplishment to even design and construct simple wireframe structures from a small number of oligonucleotides [9,104]. Nowadays, large, often intricately complex, structures consisting of several hundred or more strands, which can template the positions of large collections of accessory molecules, are used for applications that range from synthetic vaccines [105] to the nanofabrication of inorganic materials and substrates [54,95,106,107,108,109], or measurements of molecular forces that are exerted by single proteins [107].

This rapid and widespread propagation of DNA nanotechnology is partially, if not largely, dependent upon the existence of user-friendly CAD software that is available to aid researchers in conceptualizing and designing structures that are specific to their needs. Ideally, the user should not have to be an expert in DNA nanoengineering in order to utilize the techniques. This level of accessibility is what enables researchers from cell biologists to synthetic chemists to apply the techniques in their own fields. It is worth mentioning that the acceptance of a new technology is not only based on its usefulness, but also on the ease of use [110]. One of the reasons that subfields, like DNA-enabled nano-plasmonics, have gained rapid prominence is that the experts in those fields can embrace and successfully use the different techniques of DNA nano-fabrication, often DNA origami, to answer some of their most fundamental questions. Therefore, we suggest that robust and easy-to-use software provides that key inflexion point.

An illustrative example is a comparison between the widespread success of DNA origami and the slower progress of the arguably more powerful technique of DNA bricks. The rapid advancement of DNA origami following its 2006 introduction to the world [12] was quickly aided by the 2009 releases of Tiamat and Cadnano [39,41], and the subsequent development of many further software packages to account for different design strategies. Conversely, available CAD options for the DNA brick method are still much less advanced and battle-tested [111] and, in some cases, have depended upon the shrewd repurposing of software intended for other techniques [66], or the implementation of self-programmed scripts and commercial CAD software [50]. Therefore, it is no coincidence that the list of publications using the DNA origami approach as their central fabrication technique dwarfs those that depend upon the DNA brick approach.

Admittedly, this is akin to proposing a solution to the chicken and egg problem: factors, such as the conceptual simplicity of DNA origami and the simple fact that it preceded the DNA brick technique by six years, could play an equal role in motivating the rigorous development of applicable CAD software. Regardless of the specific factors that lead to robust software development, it is partially, if not fully, accurate to say that the development and rigorous upkeep of robust, freely-accessible, user-friendly software for applying the different methods of DNA-based nanofabrication is essential for shaping the continued advancement of the field.

We still see room for further development in the seamless integration of companion tools and modules for the analysis of structural designs directly into DNA-based CAD programs. Nevertheless, we do recognize that the computational resources that are required for any kind of detailed simulations are often unrealistic for what is available on the typical laptop or desktop used by academic researchers. One approach towards seamlessly combining design and rudimentary *in silico* validation could be direct integration of in-program portals for uploading to established online analysis tools, like Cando, MrDNA, or others. This, of course, would require close coordination between development teams, and it might also lead to the side-effect increased server load and according wait times, since integrated submission for analysis within the program would almost certainly lead to increased use. ADENITA is one very recent approach to merge most features into one software, which is a plugin for the free to use molecular design platform SAMSON [103]. ADENITA enables the integration of all widely used preexisting file formats, the use of non-DNA molecules (like proteins and aptamers), and it is compatible with oxDNA for detailed structural evaluation. While it can be used to create some de novo designs that are based on relatively simple motifs, such as single-stranded or double-crossover tiles, it is still limited as a design suite that is suitable for assisting with the creation of structures with any complexity, such as three-dimensional DNA origami or brick objects. Rather, its strength resides in its ability to span multiple platforms, and integrate the precise output files from each. Thus, we see this sort of “nanostructure-collage” approach as an important step forward towards robust and widely-encompassing DNA nano-fabrication options.

Even if successfully implemented, all of the aforementioned CAD software design and analysis tools are, of course, no substitute for tedious and rigorous experimental verification, and only give information regarding the final target structure, rather than the highly complex process by which it is formed. As numerous experimental and analytical studies have shown, folding and assembly pathways can be equally decisive for the proper formation of a particular design [65,112,113,114]. These can be highly impacted by ionic conditions in the surrounding buffer, or alternatively thermal annealing protocols implemented by careful design or brute-force screening of thermal annealing protocols [57,115].

## 6. Conclusions

In conclusion, DNA nanotechnology is one of the emerging frontiers of science and technology, and the heart of it is software to aid in the design of complex structures. The parallel development of CAD software to help scientists to implement new DNA-based fabrication strategies has worked in synergy with their advancement and wider propagation. Here, we have given a broad, and hopefully exhaustive, accounting of the different design packages that are available for designing simple structures of a few strands, up to massively complex complexes consisting of multiple DNA structures and even accessory molecules, such as proteins. Thus, we expect that our review will be the food for many researchers and scientists who want to enjoy this emerging area.

## 7. Materials and Methods

### 7.1. DNA Origami

Reverse-phase cartridge-purified staple oligonucleotides were shipped from Eurofins Genomics (Ebersberg, Germany) and then diluted to a final concentration of 50 μM in double-distilled water. A list of used oligonucleotide sequences can be found in Table S2. The 7249 nt-long M13mp18-phage-based scaffold strand was prepared and isolated, as previously described [19]. Before annealing, the scaffold was linearized and purified according to protocols described previously [98]. As a general annealing condition, 10 nM of linearized scaffold strand and 100 nM of each staple were mixed in TE buffer (10 mM Tris-HCl, 1 mM EDTA, pH 8.0 at 20 °C) containing 10 mM MgCl_2_, being heated to 90 °C for 5 min., and then annealed through a temperature decreasing ramp with −1 °C per minute from 89 °C to 20 °C.

### 7.2. AFM Imaging

For verification with atomic force microscopy, 5 μL of purified origami sample was placed onto a freshly cleaved mica surface (Plano GmbH, Wetzlar, Germany) that was fixed by hot glue onto a 15 mm metal specimen disc (Ted Pella, Inc., Redding, CA, USA). Origamis were purified by size exclusion using spin filtration columns with a molecular cutoff of 100 kDa (Amicon Ultra-0.5 Centrifugal Filter Unit, Merck, Germany). The structures were incubated for 60 s to allow for binding to the surface before being washed twice with 30 μL of the aforementioned TE buffer solution with 12.5 mM MgCl_2_ to remove any unbound DNA origami structures or other loose debris. The samples were imaged in Tapping Mode in the aforementioned buffer conditions using a NanoScope III Multimode AFM from Digital Instruments (Bruker Nano GmbH, Germany) with a silicon-nitride tip with a spring constant of k = 0.24 N/m (Bruker, SNL-10, cantilever tip C).

## Figures and Tables

**Table 1:** A comparison of the CAD software for scaffolded DNA origami and wireframe structures. “+” indicates an improvement, “−” a restriction of the software. The websites listed in this table have been last accessed on the 14th of April 2021.

CAD Software	Scope	Main Features	Website
Cadnano 1.0/2.0 (2009/2012) [39]	Lattice-based scaffolded DNA origami design	GUI allowing for design from scratch & manual manipulation of strands Lattice-based (honeycomb or square lattice) + 2.0 introduces undo button	cadnano.org/legacy cadnano.org (download)
Cadnano 2.5 (2018, beta)			github.com/cadnano/cadnano2.5 (download)
scadnano (2020) [40]	Lattice-based scaffolded DNA origami design	Similar to Cadnano script based online tool	scadnano.org (direct use via browser)
Tiamat (2009) [41]	Lattice and scaffold free DNA nanostructure design	+ No geometrical constrains + corrects for: secondary structures, repetitions and GC-content − needs manual adjustment	yanlab.asu.edu/Resources.html (download)
vHelix (2015) [24]	Automated 3D wireframe DNA origami design	Automated 3D wireframe design + Scaffold automatically transverses trough every edge evenly − Restricted to designs equivalent to a sphere	vhelix.net (download, req. Maya)
DAEDALUS (2016) [28]	Fully automated 3D wireframe origami design	Automated 3D wireframe origami design + No geometrical restriction to a sphere + designs stable at low salt − no GUI	daedalus-dna-origami.org (download, req. Maya)
PERDIX (2019) [45]	Fully automated 2D wireframe origami design	Automated 2D wireframe origami design + Arbitrary large 2D constructs − no GUI	perdix-dna-origami.org (download, req. Maya)
TALOS (2019) [46]	Fully automated 3D wireframe origami design (higher stability)	Automated 3D wireframe origami design + Increased mechanical stability due to six-helix edges − Material intensive, requires high salt conc.	talos-dna-origami.org (download, req. Maya)
METIS (2019) [47]	Fully automated 2D wireframe origami design (higher stability)	Automated 2D wireframe origami design + Increased mechanical stability due to six-helix edges −Material intensive, requires high salt conc. − no GUI	metis-dna-origami.org (download, req. Maya)
ATHENA (2020) [48]	Fully automated 2D & 3D wireframe origami design	Combines all features of DEADALUS, PERDIX, TALOS, METIS in an interactive GUI	github.com/lcbb/Athena (download, req. Maya)

**Table 2:** Comparison of the CAD software for DNA tiles and bricks. “+” indicates an improvement, “−” a restriction of the software. The websites listed in this table have been last accessed on the 14th of April 2021.

CAD Software	Scope	Main Features	Website
DNA Pen (2013) [67]	2D tile-based DNA designs	Free hand drawn or digitalized 2D design + Automatic inclusion of poly-T chains to prevent base stacking − only planar structures	guptalab.org/dnapen (download)
3DNA (2014) [79]	3D tile-based DNA designs	Digitalized 3D design + Allows for arbitrarily large structures + Accounts for GC content & Hamming distance	guptalab.org/3dna/index.html (download)
Hex-tiles (2019) [69]	2D Triangulated Wireframe Structures using DNA Tiles	Triangulated 2D Wireframe Structures without a scaffold + Allows for arbitrarily large structures + Rolled up sheets resemble 3D hollow tubes + physiological salt conditions	github.com/tls-dna/hex-tiles (download)

**Table 3:** Comparison of the CAD software for the analysis of DNA nanostructures. The websites listed in this table have been last accessed on the 14th of April 2021.

CAD Software	Scope	Main Features	Website
UNAfold (2008) [87]	DNA & RNA folding and hybridization prediction	Continuation of Mfold	www.unafold.org (download)
NUPACK (2010) [88]	DNA folding and hybridization prediction	Suitable for multiple strand analysis	nupack.org (download & direct use via browser)
ViennaRNA Package 2.0 (2011) [89]	RNA secondary structure prediction	RNA secondary structure prediction	www.tbi.univie.ac.at/RNA/#download (download)
CanDo (2011) [90]	2D & 3D modeling of DNA nanostructures	Finite element modeling framework for DNA origami assemblies input: caDNAno or Tiamat	cando-dna-origami.org (submission via browser)
oxDNA/oxDNA2 (2015) [97] oxView (2020) [99]	2D & 3D Coarse-grained modelling of DNA & RNA assemblies	Coarse-grained modelling of DNA/RNA for DNA origami assemblies Includes Monte Carlo and Molecular Dynamics simulations Easy to visualize via browser-based oxView	dna.physics.ox.ac.uk/index.php (download) sulcgroup.github.io/oxdna-viewer (direct use via browser)
TacoxDNA (2019) [100]	Web-based interface for converting common formats of DNA structures	input: XYZ coordinate file, cadnano, Tiamat, CanDo, oxDNA, PDB output: oxDNA, PDB	tacoxdna.sissa.it (direct use via browser)
MrDNA (2019) [101]	Fast analysis of DNA nano-structures with high resolution	Faster prediction of low- & high-resolution models at Near-Atomic Resolution Predicts 3D shape & equilibrium properties input: cadnano, vHelix, DAEDALUS, CanDo, oxDNA, PDB	gitlab.engr.illinois.edu/tbgl/tools/mrdna (download)
Adenita (2020) [103]	Universal approach for the design and/or analyisis of DNA nano-structures	Combines several previous approaches and also other molecular structures input: cadnano, vHelix, DAEDALUS	samson-connect.net/element/dda2a078-1ab6-96ba-0d14-ee1717632d7a.html (download, req. SAMSON)
SNUPI (2021) [102]	Rapid analysis of DNA Origami structures with high resolution	Rapid analysis due to a multiscale analysis framework Predicts 3D shape, equilibrium dynamic properties & mechanical rigidity input: cadnano	github.com/SSDL-SNU/SNUPI (download)

## Supplementary Materials

The following are available online. Table S1: Overview of the software covered in this review as well as their scope, main features and their website. “+” indicates an improvement, “−” a restriction of the software. The websites listed in this table have been last accessed on the 14th of April 2021; Table S2: List of used oligonucleotide sequences for AFM imaging shown in Figure 9.

## Abbreviations

The following abbreviations are used in this manuscript:

AFM	Atomic Force Microscope
CAD	Computer-assisted Design
GUI	Graphical-user Interface
TEM	Transmission Electron Microscopy
