# Supplementary material for: The Art of Designing DNA Nanostructures with CAD Software
Source: Molecules. 2021 Apr 15;26(8):2287. doi: 10.3390/molecules26082287 (PMC8071251; doi:10.3390/molecules26082287)
Supplement: Supplementary file 1 [file molecules-26-02287-s001.zip › molecules-1152663-supplementary.pdf]

Supplementary Meterials

Table S1: Overview of the software covered in this review as well as their scope, main features and their website. "+" indicates an improvement, "-" a restriction of the software. The websites listed in this table have been last accessed on the 14th of April 2021.

CAD Software	Scope	Main Features	Website
**Vienna RNA** (1994, inactive) [1]	Secondary structures of RNA strands	Early tool to predict secondary RNA structures	-
**Mfold** (2003, inactive) [2]	Secondary structures of RNA & DNA strands	Early tool to predict secondary RNA/DNA structures	-
**GIDEON** (2006, inactive) [3]	*de novo* design of 3D DNA nanostructures	GUI for the design of DNA nanostructures<br>Kink & Hairpin relaxation	-
**SARSE** (2008, inactive) [4]	Lattice-based DNA origami design	Semi-automated DNA origami design	sourceforge.net/projects/sarse (download, historical reference)
**UNIQUMER3D** (2009, inactive) [5]	*de novo* design of 3D DNA nanostructures	3D modelling tool for DNA constructs	-
Scaffold-based			
**Cadnano 1.0 /2.0** (2009/2012) [6]	Lattice-based scaffolded DNA origami design	GUI allowing for design from scratch & manual manipulation of strands<br>Lattice-based (honeycomb or square lattice)<br>+ 2.0 introduces undo button	cadnano.org/legacy<br>cadnano.org<br>(download)
**Cadnano 2.5** (2018)			github.com/cadnano/cadnano2.5 (download)
**scadnano** (2020) [7]	Lattice-based scaffolded DNA origami design	Similar to Cadnano<br>script based online tool	scadnano.org<br>(direct use via browser)
**Tiamat** (2009) [8]	Lattice and scaffold free DNA nanostructure design	+ No geometrical constrains<br>+ corrects for: secondary structures, repetitions and GC-content<br>- needs manual adjustment	yanlab.asu.edu/Resources.html (download)
**vHelix** (2015) [9]	Automated 3D wireframe DNA origami design	Automated 3D wireframe design<br>+ Scaffold automatically transverses trough every edge evenly<br>- Restricted to designs equivalent to a sphere	vhelix.net<br>(download, req. Maya)
**DAEDALUS** (2016) [10]	Fully automated 3D wireframe origami design	Automated 3D wireframe origami design	daedalus-dna-origami.org<br>(download, req. Maya)

		+ No geometrical restriction to a sphere<br>+ designs stable at low salt<br>- no GUI	
**PERDIX**<br>(2019) [11]	Fully automated 2D wireframe origami design	Automated 2D wireframe origami design<br>+ Arbitrary large 2D constructs<br>- no GUI	perdix-dna-origami.org<br>(download, req. Maya)
**TALOS**<br>(2019) [12]	Fully automated 3D wireframe origami design (higher stability)	Automated 3D wireframe origami design<br>+ Increased mechanical stability due to six-helix edges<br>- Material intensive, requires high salt conc.	talos-dna-origami.org<br>(download, req. Maya)
**METIS**<br>(2019) [13]	Fully automated 2D wireframe origami design (higher stability)	Automated 2D wireframe origami design<br>+ Increased mechanical stability due to six-helix edges<br>- Material intensive, requires high salt conc.<br>- no GUI	metis-dna-origami.org<br>(download, req. Maya)
**ATHENA**<br>(2020) [14]	Fully automated 2D & 3D wireframe origami design	Combines all features of DEADALUS, PERDIX, TALOS, METIS in an interactive GUI	github.com/lcbb/Athena<br>(download, req. Maya)
Tile-based			
**DNA Pen**<br>(2013) [15]	2D tile-based DNA designs	Free hand drawn or digitalized 2D design<br>+ Automatic inclusion of poly-T chains to prevent base stacking<br>- only planar structures	guptalab.org/dnapen<br>(download)
**3DNA**<br>(2014) [16]	3D tile-based DNA designs	Digitalized 3D design<br>+ Allows for arbitrarily large structures<br>+ Accounts for GC content &Hamming distance	guptalab.org/3dna/index.html<br>(download)
**Hex-Tiles**<br>(2019) [17]	2D Triangulated Wireframe Structures using DNA Tiles	Triangulated 2D Wireframe Structures without a scaffold<br>+ Allows for arbitrarily large structures<br>+ Rolled up sheets resemble 3Dhollow tubes<br>+ physiological salt conditions	github.com/tls-dna/hex-tiles(download)

Analytical tools			
**UNAfold** (2008) [18]	DNA & RNA folding and hybridization prediction	Continuation of Mfold	www.unafold.org (download)
**NUPACK** (2010) [19]	DNA folding and hybridization prediction	Suitable for multiple strand analysis	nupack.org (download & direct use via browser)
**ViennaRNA Package 2.0** (2011) n[20]	RNA secondary structure prediction	RNA secondary structure prediction	www.tbi.univie.ac.at/RNA/#download (download)
**CanDo** (2011) [21]	2D & 3D modeling of DNA nanostructures	Finite element modeling framework for DNA origami assemblies<br>input: caDNAno or Tiamat	cando-dna-origami.org (submission via browser)
**oxDNA/oxDNA2** (2015) [22]<br>**oxView** (2020) [23]	2D & 3D Coarse-grained modelling of DNA & RNA assemblies	Coarse-grained modelling of DNA/RNA for DNA origami assemblies<br>Includes Monte Carlo and Molecular Dynamics simulations<br>Easy to visualize via browser-based oxView	dna.physics.ox.ac.uk/index.php (download)<br>sulcgroup.github.io/oxdna-viewer (direct use via browser)
**TacoxDNA** (2019) [24]	Web-based interface for converting common formats of DNA structures	input: XYZ coordinate file, cadnano, Tiamat, CanDo, oxDNA, PDB<br>output: oxDNA, PDB	tacoxdna.sissa.it (direct use via browser)
**MrDNA** (2019) [25]	Fast analysis of DNA nano-structures with high resolution	Faster prediction of low- & high-resolution models at Near-Atomic Resolution<br>Predicts 3D shape & equilibrium properties<br>input: cadnano, vHelix, DAEDALUS, CanDo, oxDNA, PDB	gitlab.engr.illinois.edu/tbgl/tools/mrdna (download)
**Adenita** (2020) [26]	Universal approach for the design and/or analyisis of DNA nano-structures	Combines several previous approaches and also other molecular structures<br>input: cadnano, vHelix, DAEDALUS	samson-connect.net/element/dda2a078-1ab6-96ba-0d14-ee1717632d7a.html (download, req. SAMSON)
**SNUPI** (2021) [27]	Rapid analysis of DNA Origami structures with high resolution	Rapid analysis due to a multiscale analysis framework<br>Predicts 3D shape, equilibrium dynamic	github.com/SSDL-SNU/SNUPI (download)

		properties & mechanical rigidity<br>input: cadnano	

Table S2: List of used oligonucleotide sequences for AFM imaging shown in figure 9.

oligo1	CAGGCAAAGCGCCATTCGCCATTCAGGCTGCGCAATCCAGCCA
oligo2	GTAACAACCCGTCGGATTCGGCCAGTGCCAAGCTTGCA
oligo3	AAAAGCCCCAAAAACAGGAGCACCGCTTCTGGTGCCGG
oligo4	ATCACCATCAATATGATATTCATCAACATTAAATGTGA
oligo5	CCTCAGAGCATAAAGCTAATATGTACCCCGGTTGATAA
oligo6	TGCGAACGAGTAGATTTAGTGAGAAAGGCCGGAGACAG
oligo7	AGCAAACTCCAACAGGTCAATTAGCAAAATTAAGCAAT
oligo8	TTGAATCCCCCTCAAATGCATATAACAGTTGATTCCCA
oligo9	ACATAACGCCAAAAGGAATGCTTCAAAGCGAACCAGAC
oligo10	GTTTAATTTCAACTTTAATTGCGGAATCGTCATAAATA
oligo11	TTGAAAGAGGACAGATGAATACCACATTCAACTAATGC
oligo12	GGCAAAAGAATACACTAAAGTAGTAAATTGGGCTTGA
oligo13	GCTTGCAGGGAGTTAAAGGTAAGGGAACCGAACTGACC
oligo14	CCAGTCACGACGTTGTAAAACGACTCCGTGGG
oligo15	GGCGATTAAGTTGGGTAACGCCAGGGGATAGG
oligo16	ACGCCAGCTGGCGAAAGGGGGATGTCGTAACC
oligo17	AAGGGCGATCGGTGCGGGCCTCTTGACGACA
oligo18	AACAAACGTTCCTGTAGCCAGCTTTCAACCGT
oligo19	TCACGTTGAACGCCATCAAAAATGAGGGTAG
oligo20	GTGCATCTTTTGTTAAATCAGCTCGCTATCAG
oligo21	GTATCGGCGTTAATATTTTGTTAAAACAAGA
oligo22	GCTTTCCGAGATTGTATAAGCAAAACTAGCAT
oligo23	GTCTGGCCGCGGATTGACCGTAATGGTTTTC
oligo24	ACCAATAGGGTGTAGATGGGCGCATGCTGCAA
oligo25	ATTAAATTGCCAGTTTGAGGGGACCGCTATT
oligo26	TTGTAAACCTCAGGAAGATCGCACCTGTTGGG
oligo27	TCTAGCTGTAAAGATTCAAAAGGGTTTGACCA
oligo28	CTATTTTTTTAAATGCAATGCCATAACCTG
oligo29	GTCATTGCGCAAGGATAAAAATTTCGCGAGCT
oligo30	GAATCGATATACTTTTGCGGGAGAATAGTAG
oligo31	GTCAATCAATCGGTTGTACCAAAAACAGGCAA
oligo32	TGTGTAGGATAAATTAATGCCGGAAATTCGC
oligo33	CTCATATATGAGAGATCTACAAAGATTTTTA
oligo34	ATTTCAACCTGAGAGTCTGGAGCAAATTCGC
oligo35	ACCCTGTAGAACGGTAATCGTAAATATTTAAA
oligo36	TTAGATACCTGGAAGTTTCATTCCTTTAAACA
oligo37	TTTAGCTATGTTTTAAATATGCAAATCAAAA
oligo38	GAAAAGGTCTTAATTGCTGAATATATAGTCAG
oligo39	TAGCATTAATAAGAGGTCATTTTTGATTAAG
oligo40	GGCAAAGAGGATTAGAGAGTACCTTCGCGTTT
oligo41	TACGGTGTATTTCGCAAATGGTCATGAGTAA
oligo42	AGCTCAACATATTTTCATTTGGGGTTAGAACC
oligo43	GCTTAGAGGGCATCAATTCTACTAAGCCTTT
oligo44	TCCTTTTGACATCCAATAAATCATACATTATG
oligo45	GTTCAGAAGGATAGCGTCCAATACCATTGTGA
oligo46	ATCAGGTCCAGAGGGGGTAATAGTGGCTCAT
oligo47	AAGCAAAGAATAGCGAGAGGCTTTGAAAAATC
oligo48	AGGAAGCCCCCTCGTTTACCAGACAACAACA
oligo49	TAATTCGATACGAGGCATAGTAAGAGTTGAGA
oligo50	TTTAGACTAACGAGAATGACCATAACTAAAG

oligo51	AAGTTTTGCTTTACCCTGACTATTAATGCTGT
oligo52	AAAACCAACGGATTGCATCAAAAAGCGGATG
oligo53	TATCATAACGAAAGACTTCAAATATTAATTGC
oligo54	ATTACCTTGAGAAACACCAGAACGACACTCAT
oligo55	TATACCATGCTCATTCAGTGAATAGCGCGAA
oligo56	TACGTTAAGATATTCATTACCCAATCATCGCC
oligo57	TTATTACACCTTCATCAAGAGTAAACCTGCT
oligo58	TTTAGGAACGGTGTACAGACCAGGCGCAGACG
oligo59	GCCCTGACATGCGATTTTAAGAACTAAAATG
oligo60	AACAAAGCGTCAGGACGTTGGGAATGCAAAAG
oligo61	AAGAACCGTAAAACGAACTAACGGGACGATA
oligo62	CTGGCTGAGGTAGAAAGATTCATCAGCAACAC
oligo63	GTAAAATACCCAGCGATTATACCAAAGGCTT
oligo64	ACTTTTTCACAACGGAGATTTGTAATCAACGT
oligo65	AGCAACGGTGTGTCGAAATCCGCGTCTTGAC
oligo66	CAGCAGCGCTTAGCCGGAACGAGGCGCATAGG
oligo67	AGTTTCGTCACCAGTACAAAACCGATATATTCGGTCGC
oligo68	CAGGCGGATAAGTGCCGTCAATAGAAAGGAACAACTAA
oligo69	TTTGATGATACAGGAGTGTGGAACCCATGTACCGTAAC
oligo70	CTCAGAGCCACCACCCTCAATTAGCGGGGTTTTGCTCA
oligo71	CACCGTAATCAGTAGCGACTCCAGTAAGCGTCATACAT
oligo72	ACAAAAGGGCGACATTCAACCGCCACCCTCAGAACCGC
oligo73	GCAATAATAACGGAATACCACCAATGAAACCATCGATA
oligo74	ACACCCTGAACAAAGTCAGCATATGGTTTACCAGCGCC
oligo75	TTACCAACGCTAACGAGCGTACCAGAAGGAAACCGAGG
oligo76	GTTTTTATTTTCATCGTAGCATTAGACGGGAGAATTAA
oligo77	CAACATGTTCAGCTAATGCCAGCTACAATTTTATCCTG
oligo78	TCATATGCGTTATACAAATCACTCATCGAGAACAAGCA
oligo79	AAAATCTCGTTTCAGCGGAGTGAGGAGAGGGT
oligo80	CTTTAATTGTATGGGATTTTGCTGTGTATCA
oligo81	CGAGGTGATCGTCTTTCCAGACGTGCCACCCT
oligo82	ATAGTTGCCTCATAGTTAGCGTAACACCCTC
oligo83	CCACGCATACTACAACGCCTGTAGGATAGCAA
oligo84	TGATATAACAAGAGAAGGATTAGGGAGCCGCC
oligo85	CCGTACTAACATGAAAGTATTAAGCCAGCAT
oligo86	CAGAACCGCCCCCTGCCTATTTCGGACGATTG
oligo87	AGAGCCACTGAGTAACAGTGCCCGATCCTCA
oligo88	GCCCAATAACTGGTAATAAGTTTTTCTCTGAA
oligo89	AGACTCCTGTATAGCCCGGAATAGAAACAAC
oligo90	TATTCTGACAGGAGGTTTAGTACCTAGTAAAT
oligo91	AGTTAATGCCACCCTCAGAACCGCCGATCTA
oligo92	CAGTGCCTCACCCTCATTTTCAGGCATTCCAC
oligo93	ACCAGAACACCGCCTCCCTCAGAGCCGATTGA
oligo94	TGACAGGCAAAATCACCGGAACCAAATTATT
oligo95	GCCTTGATCCCCTTATTAGCGTTTCACCGACT
oligo96	TTAAAGCCGCGCGTTTTCATCGGCGCAAAAT
oligo97	TTTACCGTAGAATCAAGTTTGCCTCAAGGCCG
oligo98	CCACCGGACACCACCAGAGCCGCCGAGGCTG
oligo99	TTCATAATAGGTTGAGGCAGGTCAGAACCTAT
oligo100	GTCATAGCATTCACAAACAAATAATATAAAC
oligo101	AGACTGTAAGAATGGAAAGCGCAGAACGGGGT
oligo102	GGGAGGGACAATCAATAGAAAATTAGGGTAAT
oligo103	CATTAAAAAAGACACCACGGAATCCCACAAG
oligo104	TGAGCCATTACATAAAGGTGGCAAGAGCAAGA
oligo105	CACCAGTAACGCAGTATGTTAGCATTACCGA
oligo106	GAAACGTCCAAAAGAACTGGCATGGATAGCCG

oligo107	TTTTGTCAAGGTAAATATTGACGGAGAGCCA
oligo108	AGAAACGCGGTGAATTATCACCGTGCCATCTT
oligo109	AAAATACATTGGGAATTAGAGCCAATTTTCG
oligo110	TCCTTATTGCACCATTACCATTAGTTAGCGTC
oligo111	TGAGCGCTTAAAAACAGGGAAGCGGAATCATT
oligo112	AATTGAGGAAAATAGCAGCCTTTAGATATAG
oligo113	AACAATGATAAGAAACGATTTTTTACGCGAGG
oligo114	AGCCCTTTTAAACAGCCATATTATCGGGAGG
oligo115	AACAAAGTTCTTTCCAGAGCCTAAGTTGCTAT
oligo116	GAATAACAAATATCAGAGAGATAAAAGTTTA
oligo117	TCAAAAATTTAAGCCCAATAATAACATATAAA
oligo118	AATCCAAAAATAGCAATAGCTATCAACGTAG
oligo119	TTACAAAATTAAGAAAAGTAAGCAATTAAGAC
oligo120	ACCGCGCCATTAAACCAAGTACCGTCTTACCA
oligo121	AAGGCTTGCTGTCTTTCCTTATCGGGCTTAA
oligo122	CGTTTTAGTTTACGAGCATGTAGACGCCAACA
oligo123	TTTTGAAGCCTGAACAAGAAAAATGAGCCAG
oligo124	TTTGCACCAGAACGCGCCTGTTTACAAAAGGT
oligo125	GAACGGGTCAATAGCAAGCAAATCACAGAGA
oligo126	AATAATCGATCCGGTATTCTAAGAGTTTAACG
oligo127	CATCCTAACGAACCTCCCGACTTGTTATCCC
oligo128	AGATAAGTCCTTAAATCAAGATTATTTGCCAG
oligo129	ATGTGAGTGAATAACCTTGTACTAGAAAAAGCCTGTTT
oligo130	CAATAACGGATTCGCCTGATTAGGTTGGGTTATATAAC
oligo131	TCAGATGATGGCAATTCATGGAAACAGTACATAAATCA
oligo132	ATTAGAGCCGTCAATAGATAGTACCTTTTACATCGGGA
oligo133	TATTAACACCGCCTGCAACAATTATCATCATATTCCTG
oligo134	AAAGGGACATTCTGGCCAATTAGGAGCACTAACAACTA
oligo135	AATAACATCACTTGCCTGATAAAACAGAGGTGAGGCGG
oligo136	CGTATAACGTGCTTTCCTCCACCAGTCACACGACCAGT
oligo137	GGAGCCCCCGATTTAGAGCGTAGCAATACTTCTTTGAT
oligo138	TAGCCCGAGATAGGGTTGATACTATGGTTGCTTTGACG
oligo139	TTTGCGTATTGGGCGCCAGAGCACTAAATCGGAACCCT
oligo140	TTCTTTTCGCTGCATTAATGAATCGGCCAACGCGCGGGGAGAG
oligo141	GAAATTGTTATCCGCTCACAAATCCCTTATAAATCAAA
oligo142	CTTTGACCCGTAATGCCACTACGATCACGTTG
oligo143	ACAAAGTATGAGGAAGTTTCCATAAAGGAGC
oligo144	TGATAAATCTACAGAGGCTTTGAGCTTGCTTT
oligo145	CCATGTTAAAAGACAGCATCGGAAGATACCG
oligo146	GTCAATCACCGCTTTTGCGGGATCACCATCGC
oligo147	ATTGCGAATAATAATTTTTAGGCACCAACCTAAAACGA
oligo148	TTTCAACACAAAAAAAAGGCTCCATAAACGG
oligo149	GAATTTTCTGTATCGGTTTATCAGGACTAAAG
oligo150	AAGTTTTGATTTCTTAAACAGCTTCGAGGGT
oligo151	AGACAGCCGCCGACAATGACAACAGTCACCCT
oligo162	AATCGTCGAATTACCTTTTTTAATCAATATAA
oligo163	TCCTTGACAAAATTAATTACATTTGAATAAT
oligo164	GACGCTGAAAAAGAAGATGATGAACAAAATTA
oligo165	AAAATCATGAGGCGAATTATTCATAAATTGC
oligo166	CCTCCGGCTTGCTTTGAATACCAAATGAATAT
oligo167	TTCATTTGCTATTAATTAATTTTCGTTAAAT
oligo168	CAAGAAAAAAACATAGCGATAGCTGTTTGAAA
oligo169	ACCTGAGCGAAGAGTCAATAGTGAGTTAATT
oligo170	ATCGCGCAAGGTCTGAGAGACTACGAACGCGA
oligo171	TCCTGATTCCACCAGAAGGAGCGGAGTGCCAC
oligo172	GGAAGGGAGTAACATTATCATTTAATCTAAA

oligo173	TTTGCACGTTTGCCCGAACGTTATTATCAAAC
oligo174	GTAGATTTTACAAACAATTCGACAGCAAATC
oligo175	ACAGTAACAATACATTTGAGGATTGTTATCTA
oligo176	CAAAGAAAGTTTGGATTATACTTCTAACAAT
oligo177	AAAGTTTGTTAGAACCTACCATATACAAACAT
oligo178	TTAAATCCTAAAACAGAAATAAAGTTCAATT
oligo179	TTAGACTTTCAGGTTTAACGTCAGGTTACAAA
oligo180	GCTGAGAGAACCACCAGCAGAAGAGTAGAAGA
oligo181	GCATCACCCTAAAACATCGCCATAATATCCA
oligo182	CCTCAATCGGCTATTAGTCTTTAAGCAACAGG
oligo183	AACAGTTGAGAATACGTGGCACAGATTTTGA
oligo184	AAATATCTCAGAGATAGAACCCTTTTTACATT
oligo185	ACCGAACGCCAGCAGCAAATGAAATGCGGAA
oligo186	CTGATAGCCTTGCTGAACCTCAAATAATTTTA
oligo187	TTTTGAATAATATCTGGTCAGTTGACTCGTA
oligo188	AAAGCGTAAAAGGAATTGAGGAAGTAGAAGTA
oligo189	ACTCAAACACGCAAATTAACCGTTTTGACGGG
oligo190	GAACAATTGAGGCCACCGAGTAAAGGAAGGG
oligo191	AAAAACGCGCCAGAATCCTGAGAATAGGGCGC
oligo192	CGCTCAATCGATTAAAGGGATTTTGCGTAAC
oligo193	GGCAGATTGTTAGAATCAGAGCGGGCCGCTAC
oligo194	TGTCCATCTATCGGCCTTGCTGGTTAAAAAT
oligo195	ATAATCAGATTACCGCCAGCCATTTGCGCGAA
oligo196	AACGGTACTCATGGAAATACCTACACAATAT
oligo197	CAGGAGGCCGTCTGAAATGGATTACTGACCTG
oligo198	GAAAGCCGGGTCGAGGTGCCGTAAGGTGGTTT
oligo199	AAGAAAGCGTGAACCATCACCCAACAGCTGA
oligo200	TGGCAAGTGAAAAACCGTCTATCAAGAGAGTT
oligo201	CACCACACTTAAAGAACGTGGACTGCCCCAG
oligo202	AGGGCGCGGTGTTGTTCCAGTTTGGGTTCCGA
oligo203	TTTTTTGGGCGAACGTGGCGAGAAAAGAGTC
oligo204	GCCCACTACGAAAGGAGCGGGCGCGTGTTTTT
oligo205	CAAAGGGCGTAGCGGTCACGCTGCAGACAGG
oligo206	GTCCACTACCGCCGCGCTTAATGCGAGCTAAA
oligo207	TTGCCCTCCGCTTTCCAGTCGGGAAACCTG
oligo208	GCAGCAAGCTAACTCACATTAATTGCGTTGCG
oligo209	CAGGCGAAAGTGTAAAGCCTGGGGTGCCTAA
oligo210	AATCGGCAAATTCCACACAACATACGAGCCGG
oligo211	TCGTGCCAACCAGTGAGACGGGCAAATCAAG
oligo212	CTCACTGCTCACCGCCTGGCCCTGGGGCGATG
oligo213	TGAGTGAGCGGTCCACGCTGGTTTCCAACGT
oligo214	AAGCATAAAATCCTGTTTGATGGTGAACAAGA
oligo215	GTATAAAGACACCGGAATCATAATCTTCTGTA
oligo216	TTGAGAAGTGTGATAAATAAGGCCCTTAGAA
oligo217	TGTAATTTTGACCTAAATTTAATGTAGATTAA
oligo218	TAATAAGATTTCAAATATATTTTAATTTATC
oligo219	AAAGTAATCCAATCGCAAGACAAACTTTTTAA
oligo220	AAGAATAACCAACGCTCAACAGTAATTCCAA
oligo221	TACCGACCTCGCCATATTTAACAAAACCAATC
oligo222	TCATCTTCAGGCAGAGGCATTTTCAATATCC
oligo223	GAAAACTTGAATATAAAGTACCGATCAACAAT
oligo224	TGTAAATGCTGATGCAAATTCTGTCCAGACGACGACAA

References:

[1] Hofacker, I.L.; Fontana, W.; Stadler, P.F.; Bonhoeffer, L.S.; Tacker, M.; Schuster, P. Fast folding and comparison of RNA secondary structures. *Monatsh Chem* **1994**, 125, 167–188. doi:10.1007/BF00818163.

[2] Zuker, M. Mfold web server for nucleic acid folding and hybridization prediction. *Nucleic Acids Res.* **2003**, 31, 3406–3415. doi:10.1093/nar/gkg595.

[3] Birac, J.J.; Sherman, W.B.; Kopatsch, J.; Constantinou, P.E.; Seeman, N.C. Architecture with GIDEON, a program for design in structural DNA nanotechnology. *J Mol Graph Model* **2006**, 25, 470–480. doi:10.1016/j.jmgm.2006.03.005.

[4] Andersen, E.S.; Dong, M.; Nielsen, M.M.; Jahn, K.; Lind-Thomsen, A.; Mamdouh, W.; Gothelf, K.V.; Besenbacher, F.; Kjems, J. DNA Origami Design of Dolphin-Shaped Structures with Flexible Tails. *ACS Nano* **2008**, 2, 1213–1218. doi:10.1021/nn800215j.

[5] Zhu, J.; Wei, B.; Yuan, Y.; Mi, Y. UNIQUIMER 3D, a software system for structural DNA nanotechnology design, analysis and evaluation. *Nucleic Acids Res.* **2009**, 37, 2164–2175. doi:10.1093/nar/gkp005.

[6] Douglas, S.M.; Marblestone, A.H.; Teerapittayanon, S.; Vazquez, A.; Church, G.M.; Shih, W.M. Rapid prototyping of 3D DNA-origami shapes with caDNAno. *Nucleic Acids Res.* **2009**, 37, 5001–5006. doi:10.1093/nar/gkp436.

[7] Doty, D.; Lee, B.L.; Stérin, T. scadnano: A browser-based, scriptable tool for designing DNA nanostructures. *arXiv:2005.11841 [cs,q-bio]* **2020**. arXiv: 2005.11841.

[8] Williams, S.; Lund, K.; Lin, C.; Wonka, P.; Lindsay, S.; Yan, H. Tiamat: A Three-Dimensional Editing Tool for Complex DNA Structures. DNA Computing; Goel, A.; Simmel, F.C.; Sosík, P., Eds. Springer, **2009**, Lecture Notes in Computer Science, pp. 90–101. event-place: Berlin, Heidelberg, doi:10.1007/978-3-642-03076-5_8.

[9] Benson, E.; Mohammed, A.; Gardell, J.; Masich, S.; Czeizler, E.; Orponen, P.; Högberg, B. DNA rendering of polyhedral meshes at the nanoscale. *Nature* **2015**, 523, 441–444. doi:10.1038/nature14586.

[10] Veneziano, R.; Ratanalert, S.; Zhang, K.; Zhang, F.; Yan, H.; Chiu, W.; Bathe, M. Designer nanoscale DNA assemblies programmed from the top down. *Science* **2016**, 352, 1534–1534. doi:10.1126/science.aaf4388.

[11] Jun, H.; Zhang, F.; Shepherd, T.; Ratanalert, S.; Qi, X.; Yan, H.; Bathe, M. Autonomously designed free-form 2D DNA origami. *Sci. Adv.* **2019**, 5, eaav0655. doi:10.1126/sciadv.aav0655.

[12] Jun, H.; Shepherd, T.R.; Zhang, K.; Bricker, W.P.; Li, S.; Chiu, W.; Bathe, M. Automated Sequence Design of 3D Polyhedral Wireframe DNA Origami with Honeycomb Edges. *ACS Nano* **2019**, 13, 2083–2093. doi:10.1021/acsnano.8b08671.

[13] Jun, H.; Wang, X.; Bricker, W.P.; Bathe, M. Automated sequence design of 2D wireframe DNA origami with honeycomb edges. *Nat. Commun.* **2019**, 10, 5419. doi:10.1038/s41467-019-13457-y.

[14] Jun, H.; Wang, X.; Bricker, W.P.; Jackson, S.; Bathe, M. Rapid Prototyping of Wireframe Scaffolded DNA Origami using ATHENA. *bioRxiv* **2020**, p. 2020.02.09.940320. doi:10.1101/2020.02.09.940320.

[15] Goyal A, Limbachiya D, Gupta S K, Joshi F, Pritmani S, Sahai A and Gupta M K 2013 DNA Pen: A Tool for Drawing on a Molecular Canvas *arXiv:1306.0369 [cs]*

[16] Gupta, S.K.; Joshi, F.; Limbachiya, D.; Gupta, M.K. 3DNA: A Tool for DNA Sculpting. *arXiv:1405.4118 [cs]* **2014**. arXiv: 1405.4118.

[17] Matthies, M.; Agarwal, N.P.; Schmidt, T.L. Design and Synthesis of Triangulated DNA Origami Trusses. *Nano Lett.* **2016**, 16, 2108–2113. doi:10.1021/acs.nanolett.6b00381.

[18] Markham, N.R.; Zuker, M. UNAFold: software for nucleic acid folding and hybridization. *Methods Mol. Biol.* **2008**, 453, 3–31. doi:10.1007/978-1-60327-429-6_1.

[19] Zadeh, J.N.; Steenberg, C.D.; Bois, J.S.; Wolfe, B.R.; Pierce, M.B.; Khan, A.R.; Dirks, R.M.; Pierce, N.A. NUPACK: Analysis and design of nucleic acid systems. *J. Comput. Chem* **2011**, 32, 170–173. doi:https://doi.org/10.1002/jcc.21596.

[20] Lorenz, R.; Bernhart, S.H.; Höner zu Siederdissen, C.; Tafer, H.; Flamm, C.; Stadler, P.F.; Hofacker, I.L. ViennaRNA Package 2.0. *Algorithms Mol. Biol.* **2011**, 6, 26. doi:10.1186/1748-7188-6-26.

[21] Castro, C.E.; Kilchherr, F.; Kim, D.N.; Shiao, E.L.; Wauer, T.; Wortmann, P.; Bathe, M.; Dietz, H. A primer to scaffolded DNA origami. *Nat. Methods* **2011**, 8, 221–229. doi:10.1038/nmeth.1570.

[22] Snodin, B.E.K.; Randisi, F.; Mosayebi, M.; Šulc, P.; Schreck, J.S.; Romano, F.; Ouldridge, T.E.; Tsukanov, R.; Nir, E.; Louis, A.A.; Doye, J.P.K. Introducing improved structural properties and salt dependence into a coarse-grained model of DNA. *J. Chem. Phys.* **2015**, 142, 234901. doi:10.1063/1.4921957.

[23] Poppleton, E.; Bohlin, J.; Matthies, M.; Sharma, S.; Zhang, F.; Šulc, P. Design, optimization and analysis of large DNA and RNA nanostructures through interactive visualization, editing and molecular simulation. *Nucleic Acids Res.* **2020**, 48, e72–e72. doi:10.1093/nar/gkaa417.

[24] Suma, A.; Poppleton, E.; Matthies, M.; Šulc, P.; Romano, F.; Louis, A.A.; Doye, J.P.K.; Micheletti, C.; Rovigatti, L. TacoxDNA: A user-friendly web server for simulations of complex DNA structures, from single strands to origami. *J. Comput. Chem* **2019**, 40, 2586–2595. doi:https://doi.org/10.1002/jcc.26029.

[25] Maffeo, C.; Aksimentiev, A. MrDNA: a multi-resolution model for predicting the structure and dynamics of DNA systems. *Nucleic Acids Res.* **2020**, 48, 5135–5146. doi:10.1093/nar/gkaa200.

[26] de Llano, E.; Miao, H.; Ahmadi, Y.; Wilson, A.J.; Beeby, M.; Viola, I.; Barisic, I. Adenita: interactive 3D modelling and visualization of DNA nanostructures. *Nucleic Acids Res.* **2020**, 48, 8269–8275. doi:10.1093/nar/gkaa593.

[27] Lee, J.Y.; Lee, J.G.; Yun, G.; Lee, C.; Kim, Y.J.; Kim, K.S.; Kim, T.H.; Kim, D.N. Rapid Computational Analysis of DNA Origami Assemblies at Near-Atomic Resolution. *ACS Nano* **2021**, 15, 1002–1015. doi:10.1021/acsnano.0c07717.
